# The Relation between *Helicobacter pylori* Infection and Acute Bacterial Diarrhea in Children

**DOI:** 10.1155/2014/191643

**Published:** 2014-02-19

**Authors:** Maryam Monajemzadeh, Ata Abbasi, Parin Tanzifi, Sahar Taba Taba Vakili, Heshmat Irani, Leila Kashi

**Affiliations:** ^1^Clinical and Surgical Pathology, Department of Pathology, Children Medical Center Hospital, Tehran University of Medical Sciences, Tehran 14161351, Iran; ^2^Pediatric Infectious Disease Research Center, Children Medical Center Hospital, Tehran University of Medical Sciences, Tehran 14161351, Iran; ^3^Department of Pathology, Tehran University of Medical Science, Tehran 1439665663, Iran; ^4^Department of Pathology, Children Medical Center Hospital, Tehran University of Medical Science, Tehran 14161351, Iran; ^5^Department of Gastroenterology, Tehran University of Medical Science, Tehran 1419733141, Iran; ^6^Children Medical Center Hospital, Tehran University of Medical Sciences, Tehran 14161351, Iran

## Abstract

*Background*. *H. pylori* infection leads to chronic gastritis in both children and adults. But recently, there are arising theories of its protective effect in diarrheal diseases. *Aim*. To explore the prevalence of *H. pylori* infection in children with bacterial diarrhea and compare it with healthy controls. *Patients and Methods*. Two matched groups consisted of 122 consecutive children, aged 24–72 months old, with acute bacterial diarrhea, who had Shigellosis (*N* = 68) and Salmonellosis (*N* = 54) as patients group and 204 healthy asymptomatic children as control group enrolled in this study. *Results*. The prevalence of *H. pylori* infection in healthy control children was significantly higher than in patients group, (odds ratio = 3.6, 95% CI: 1.33–9.5, *P* = 0.007). In our study, only 2/54 *Salmonella* infected patients and 3/68 of Shigellosis had evidence of *H. pylori* infection, while normal control children had 27/204 infected individuals. *Conclusion*. *H. pylori* infection may play a protective role against bacterial diarrhea in children. So it is important to consider all of the positive and negative aspects of *H. pylori* infection before its eradication.

## 1. Introduction

*Helicobacter pylori* (*H. pylori*) is one of the most important factors in the gastroduodenal diseases. The infection is most commonly acquired in early childhood and leads to chronic gastritis in both children and adults and is the leading cause of peptic ulcer disease in humans [1–4]. It is a challenging matter for many physicians due to lack of knowledge about its life cycle and low rate of bacterial eradication. *H. pylori* has been shown to play a major role in the pathogenesis of gastric atrophy, chronic diarrhea, and growth retardation in children, intestinal metaplasia, dysplasia, and the development of gastric carcinoma and lymphoma subsequently [5–7]. Besides, some benefits are also noted such as reducing prevalence of esophageal adenocarcinoma by decreasing the periods of gastroesophageal reflux disease [8]. Fecal-oral or oral-oral routes are the main candidate ways of its transmission, although much is unknown in this regard [9].

Gastroenteritis is another important disease in children caused by a variety of bacterial and viral agents and the exact responsible organisms are different from one area to another but generally, *E. coli*, *Shigella*, and *Salmonella* are the most common enteric bacterial pathogens [10, 11].

Recently, there are arising theories of protective effect of *H. pylori* infection on diarrheal diseases, showing the low prevalence of diarrhea in children infected by *H. pylori* [12–14], although there is still a living debate about it. On the other hand, it has been shown that *H. pylori* infection is associated with *Vibrio cholerae* and *Salmonella* infection possibly through hypochlorhydria resulting from acute or chronic *H. pylori* infection [9, 14].

As *Salmonella* and *Shigella* entritis and also *H. pylori* infection are common gastrointestinal problems in children, especially in developing and underdeveloped countries, we designed this study to investigate the association between *H. pylori* infection and this bacterial gastroenteritis in children.

## 2. Patients and Methods

### 2.1. Patients

The study is a case control study performed during 2009–2011 in the Children Medical Center Hospital, the major children's hospital of Iran affiliated to Tehran University of Medical Sciences, Tehran, Iran.

Two matched groups were studied as follows: (1) group of children with acute bacterial gastroenteritis due to *Salmonella* or *Shigella* consisted of 122 consecutive children (55 females and 67 males), aged 24–72 months old, 68 with Shigellosis and 54 with Salmonellosis; (2) control group consisted of two hundred and four healthy children (100 males and 104 females) with no history of diarrhea and no bacterial growth in their stool cultures. Age parameter was matched between both genders (*P* > 0.05).

Healthy children with history of diarrhea within 2 weeks before sampling and any child in each group with history of antibiotic therapy within 4 weeks prior to stool collection were excluded from study. None of the participants were treated with a proton-pump inhibitor or bismuth preparations. No underlying medical disease or immunodeficiency was detected in enrolled groups.

Personal data including age (1–3 years and 3–6 years), gender, and address was also obtained from parents using a structured questionnaire. Cases were from urban area but parents defer to get information about the exact socioeconomic level but as the hospital is governmental and located in the middle part of the city and the admitted children were from nearby areas, one can conclude that cases belonged to medium socioeconomic level. The study was approved by the Tehran University of Medical Sciences Ethics Committee.

### 2.2. Determination of *H. pylori* Status

Stool samples were collected in the hospital and immediately frozen and stored at −70°C. Solid and semisolid stool specimens were tested for *H. pylori* antigen by the GA Generic Assays GmbH kit, *H. pylori* sandwich enzyme immunoassay (Germany), in accordance with the manufacturer's instructions. In summary frozen specimens were immediately thawed and 100 mg (2-3 mm in diameter of a solid specimen) of feces was suspended thoroughly. Then the specimens were treated with anti-*H. pylori* antibody (antibiotin conjugate) and incubated at room temperature for 60 minutes. After washing, streptavidin-HRP conjugate (included in the kit) was added and the solution was incubated for 30 min at room temperature, then substrate F (included in the kit) was added, and after 15 min of incubation stop solution was added and finally the OD was read at 450 nm and interpreted according to the manufacturer's guideline.

### 2.3. Media for the Isolation of Bacteria from Stools

Bacteria from stool samples were cultivated on eight different selective agar plates in order to isolate the microorganisms. MacConkey agar was used for the detection of *E. coli*, *Salmonella*, and *Shigella* species. Thiosulfate-citrate-bile salts-sucrose (TCBS) agar was used for the detection of Vibrio species, mannitol-malt agar (MSA) for *Staphylococcus aureus*, and SS and XLD for *Shigella* and *Salmonella*. The growth pattern of each organism on the specific agar including color and also biochemical tests were also used for organism detection.

### 2.4. Statistical Analysis

The results are expressed as mean ± SD. Statistical analyses were performed using SPSS version 16.0.1 (SPSS Inc., Chicago, IL, USA). The statistical differences between proportions were determined by *χ* ^2^ analysis. Numerical data were evaluated using analysis of variance, followed by Tukey's post hoc test. *P* values less than 0.05 were considered to be statistically significant.

## 3. Results

Among 112 patients enrolled in this study, 68 had *Shigella* infection and 54 had *Salmonella* infection. The prevalence of *H. pylori* infection in control group was significantly higher than in acute bacterial diarrhea group (*P* = 0.007), in which two of fifty-four cases of *Samonella* infection and three of sixty-eight cases of Shigellosis were infected by *H. pylori* while 27 out of 204 control children were infected. The risk of positive *H. pylori* infection in control group was 3.6 (odds ratio = 3.6, 95% CI: 1.33–9.5, *P* = 0.007) times higher than bacterial infection group (Table 1). There was no statistical difference in prevalence of *H. pylori* infection in variable age groups in control versus patients groups (data not shown).

## 4. Discussion

Diarrheal disease is recognized as a globally burdensome disease, associated with considerable children's mortality and morbidity specifically in developing countries where most of the complications occur and can cause up to three million deaths per year [3, 15, 16].

The cause of diarrhea differs from one country to another and generally one can expect higher rates of bacterial infections in underdeveloped and developing countries in comparison with developed countries [15]. Several pathogenic microorganisms are incriminated as the etiologic agents of acute diarrhea such as rotavirus, adenoviruses, caliciviruses, and astroviruses, *Salmonella*, *Shigella*, enteropathogenic and enterotoxigenic *E. coli* (EPEC and ETEC, resp.), *Aeromonas*, *Plesiomonas* spp. *Campylobacter jejuni*, *Yersinia enterocolitica*, *Vibrio cholerae*, protozoa, and helminthes [17, 18].

Gastroenteritis, especially bacterial gastroenteritis, mostly occurs in developing areas where *H. pylori* infection is more prevalent and it has been proposed that there may be a common rout for both *H. pylori* and *Shigella* transmission by houseflies [3]. Previous studies have shown conflicting results on association of *H. pylori* infection with other gastrointestinal pathogens [3, 7, 9, 14].

In this study we evaluated the *H. pylori* status in children with gastroenteritis and compared it with normal controls. We focused on *Shigella* and *Salmonella* as they are the most frequent isolates from stool cultures of children in our area.

Our results revealed that the prevalence of *H. pylori* infection in healthy children was higher than in patients with bacterial diarrhea indicating that bacterial infected patients are less infected with *H. pylori*. These findings are in line with other studies showing protective role of *H. pylori* infection and reporting a reduced frequency of diarrheal illness in *H. pylori* infected children [12, 14]. It can be due to either increase in gastric acid production in individuals infected by *H. pylori* which leads to bacterial pathogens damage [19] or activation of immune system as a result of *H. pylori* infection and increased IgA production, which is fatal for enteric pathogens [20–23]. As a matter of fact, gastric acid and peptides are necessary for activation of some enteric pathogens especially that viruses and *H. pylori* infection can disrupt this process [24–27]. Previous studies have shown that children's response to *H. pylori* infection is more severe than adults so this observation may be in part due to the natural response of immune system of children.

Contradictorily, some studies have shown *H. pylori* infection to be a risk factor of some bacterial gastroenteritis [28, 29]. According to the fact that the pathogenesis and etiology of *H. pylori* infection are not fully understood and there are very few studies considering the relation between *H. pylori* infection and other bacterial pathogens causing gastroenteritis, further investigations, considering different aspects including *H. pylori* life cycle, products, and cytotoxins are needed. Considering lack of correlation between prevalence of *H. pylori* infection and gender or age, it can be suggested that the difference in *H. pylori* prevalence in the two examined groups would be due to the organism itself. This hypothesis was previously noted by Perry et al. [13].

Our study has some limitations, such as having rather small number of enrolled patients in this study which may to some extent weaken our results. Another issue is about the method. Parenthetically, it should be mentioned that the specificity and sensitivity of the used kit were 100% and 91%, respectively, according to its manual. In the literature review, we found some conflicting data about this test but many of the articles indicated it as a good alternative in children [30–34].

There are different complications in *H. pylori* infection such as gastritis, gastric lymphoma, and carcinoma, but in contrast *H. pylori* infection decreases the risk of esophageal adenocarcinoma [6, 35, 36]. There are many studies showing association or protective role of *H. pylori* infection in different disease groups, for example, reducing Barrett's esophagitis [37], decreasing risk of esophageal cancer [38], decreasing risk of gastric cardiac cancer [39], or decreasing the bleeding risk of esophageal varices in cirrhotic patients [40]. According to our findings, *H. pylori* infection may be candidate agent which plays a protective role in gastroenteritis in children. So it is important to consider all of the positive and negative aspects of *H. pylori* infection before its eradication; however, because of the current debate and considering the fact that knowledge about *H. pylori* life cycle and its pathogenesis is not explicitly defined more comprehensive research is recommended. Searching for viral gastroenteritis and determining the Cag A status of *H. pylori* by means of PCR method are also suggested.

## Figures and Tables

**Table 1:** * H. pylori* prevalence in different groups.

	HP* positive	HP negative	Total	*P* value
Bacterial diarrhea				
*Shigella *	3	65	68	
*Salmonella *	2	52	54	
Control	27	177	204	0.007

*HP: *H. pylori*.
